# *Bacillus subtilis* RNase HII Is Inefficient at Processing Guanosine Monophosphate and Damaged Ribonucleotides

**DOI:** 10.1111/mmi.70047

**Published:** 2026-01-06

**Authors:** Julianna R. Cresti, Lyle A. Simmons

**Affiliations:** ^1^ Department of Molecular, Cellular, and Developmental Biology University of Michigan Ann Arbor Michigan USA

**Keywords:** *Bacillus subtilis*, DNA damage, DNA replication, mismatches, ribonucleotides, RNase HII

## Abstract

During one round of DNA replication, nearly 2000 ribonucleoside monophosphates (rNMPs) are incorporated in place of their cognate deoxyribonucleoside monophosphates (dNMPs). Given their high rate of insertion, genomic DNA could contain rNMPs that are damaged or mismatched. Here, we test the activity of *Bacillus subtilis* and *Escherichia coli* RNase HII on canonical, mismatched, and damaged rNMPs. We show that *E. coli* RNase HII is adept at incising most rNMP variants from DNA at similar frequencies, with the exception of an oxidized rNMP, where endoribonuclease activity is sharply reduced. In contrast, *B. subtilis* RNase HII efficiently incises rAMP, rCMP, and rUMP but is inefficient at processing rGMP in both a canonical and mismatched base pair. We test damaged ribonucleotides and find that *B. subtilis* RNase HII is refractory to processing abasic and oxidized ribonucleotide lesions. Our work shows that bacterial RNase HII enzymes have different intrinsic endoribonuclease activity toward the repair of canonical, mismatched, and damaged rNMPs, demonstrating that not all rNMP errors provoke efficient resolution. Our finding that *B. subtilis* RNase HII is recalcitrant to repairing damaged rNMPs resembles what is observed for eukaryotic RNase H2 orthologs, suggesting that other repair processes are necessary to resolve damaged rNMPs.

## Graphical abstract

*Bacillus subtilis* RNase HII corrects ribonucleotide errors and mismatched ribonucleotides, but not damaged ribonucleotides. Mismatched ribonucleotides would also serve as substrates for mismatch repair while damaged ribonucleotides would be addressed through base excision repair.

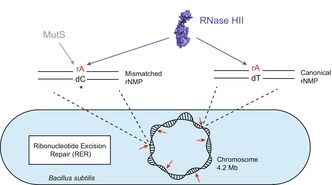

## 1 Introduction

Ribonucleotides are routinely incorporated into genomic DNA through the essential process of DNA replication (Nick McElhinny, Kumar, et al. 2010; Yao et al. 2013). It is well established that bacterial and eukaryotic replicative DNA polymerases make errors by incorporating a ribonucleoside monophosphate (rNMP) in place of its corresponding deoxyribonucleoside monophosphate (dNMP) (Yao et al. 2013; Schroeder et al. 2017; Nick McElhinny, Kumar, et al. 2010; Nick McElhinny, Watts, et al. 2010). In bacteria, it has been reported that rNMP errors occur in vitro about 2000 times per round of replication (Yao et al. 2013). These errors have been measured in vivo with numbers ranging between approximately 200 to over 2200 per round of replication across different bacterial species (Zatopek et al. 2019; Kouzminova et al. 2017). Similarly, in eukaryotes, rNMP errors can exceed 10,000 per round of replication in *Saccharomyces cerevisiae* and have been predicted to be in the millions for multicellular eukaryotes, including mice and humans (Nick McElhinny, Watts, et al. 2010; Williams and Kunkel 2014; Zhou et al. 2021). Ribonucleotide errors are often referred to as “sugar errors” because, although the nitrogenous base is correct, the ribose sugar has a hydroxyl group (‐OH) at the 2′ carbon (C2′) instead of a hydrogen atom. The 2′‐OH is reactive and has the potential to become a good nucleophile, causing phosphodiester bond cleavage (Oivanen et al. 1998) and forming a cyclic 2′,3′‐phosphate that cannot be ligated or act as a substrate for extension by a DNA polymerase (Oivanen et al. 1998). Further, ribonucleotides can be mutagenic upon removal. For example, rNMPs can be removed by topoisomerase I in eukaryotes, but this causes single‐stranded breaks and deletions (Kim et al. 2011). Likewise, in bacteria, rNMP errors can be removed using an error‐prone pathway that results in GC‐AT transitions (Schroeder et al. 2017). Given the propensity for single ribonucleotide errors to occur in vivo, it has been estimated that rNMPs represent the most frequent type of nucleotide in need of repair in genomes ranging from bacteria to humans (Nick McElhinny, Watts, et al. 2010; Schroeder et al. 2015; Williams et al. 2016; Zhou et al. 2021).

Ribonucleotides embedded in the genome are repaired through ribonucleotide excision repair (RER), which has been previously studied in *Escherichia coli* , archaea, yeast, and multicellular eukaryotes (Sparks et al. 2012; Schroeder et al. 2017; Heider et al. 2017). Although cells have many RNases, Type 2 Ribonuclease H (RNase H) enzymes, Ribonuclease HII (RNase HII) in bacteria and archaea or Ribonuclease H2 (RNase H2) in eukaryotes, initiate this process. RNase HII binds the ribonucleotide in double‐stranded DNA (dsDNA) and hydrolyzes the phosphodiester bond 5′ to the ribonucleotide (Itaya et al. 1999; Ohtani et al. 1999a). Acidic residues, together which form the DEDD motif, coordinate divalent metal cations, preferentially Mg^2+^, and facilitate hydrolysis of the backbone (Rychlik et al. 2010; Randall et al. 2017). During the canonical RER pathway in bacteria, DNA polymerase I binds the nick, removes the ribonucleotide using its 5′–3′ exonuclease activity, and substitutes the rNMP with the corresponding dNMP (Schroeder et al. 2017). RNase H2 in eukaryotes also incises the dsDNA 5′ to the rNMP (Rychlik et al. 2010). The rNMP is then removed by flap endonuclease 1 (FEN1), while DNA polymerase δ (Pol δ), aided by proliferating cell nuclear antigen (PCNA) and the clamp loader, catalyzes strand displacement synthesis (Sparks et al. 2012). Since rNMPs are incorporated at such a high frequency, redundancy in this pathway occurs—Exonuclease 1 can function in place of FEN1, and DNA polymerase ε can replace Pol δ during strand displacement synthesis and gap filling (Sparks et al. 2012). In bacteria, redundancies across repair pathways remain unclear. There is evidence in *E. coli* that nucleotide excision repair (NER) can function in the absence of RNase HII and that translesion DNA polymerases can participate in error correction, albeit increasing mutagenesis during the resynthesis step (Vaisman et al. 2013, 2014). Additional results in *E. coli* reveal that RNase HII is physically associated with RNA polymerase (RNAP), suggesting that surveillance and recognition of ribonucleotides by RNase HII is coupled to RNAP; this interaction ensures that ribonucleotides are efficiently detected in DNA during active transcription (Hao et al. 2023).

Due to the nearly 2,000 ribonucleotides incorporated into genomic DNA per round of synthesis, it is expected that some rNMPs will become damaged or otherwise modified (Randerath et al. 1992; Loeb and Preston 1986; Cheng et al. 1992; Malfatti et al. 2017). However, it remains unclear if and how cells can repair mismatched or damaged ribonucleotides. Prior work has shown that replicative DNA polymerases are poor at using their 3′–5′ “proofreading” exonuclease activity to remove properly base‐paired ribonucleotides (Williams et al. 2012). When rNMPs are paired with the incorrect complement and form a mismatch, they can serve as targets for both mismatch repair (MMR) by MutSα and RNase H2, as shown in *S. cerevisiae* (Shen et al. 2011). *E. coli* RNase HII has been shown to incise mismatched rNMPs (Malfatti et al. 2019). In one study, *Pyrococcus abyssi* RNase HII was unable to incise a rG:dA mismatch (Malfatti et al. 2019), while in another study the same protein was moderately active on the same substrate (Reveil et al. 2023). Therefore, on balance, some Type 2 RNase H enzymes are at least capable of endoribonuclease activity on mismatched rNMPs, while specific enzymes, like *E. coli* RNase HII, have been shown to prefer mismatched rNMP substrates (Malfatti et al. 2019). Moreover, given the high rate of misincorporation, damaged rNMPs, including abasic sites (rOH) and lesions generated from oxidative damage (i.e., r8oG), would be expected to occur in most cells during normal growth (Malfatti et al. 2017, 2019). Interestingly, rOH and r8oG do not serve as substrates for eukaryotic or archaeal RNase H2 but are instead repaired by apurinic/apyrimidinic endonuclease 1 (APE1) (Malfatti et al. 2017, 2019). In contrast, *E. coli* RNase HII has been reported to incise both lesions in vitro (Malfatti et al. 2019). To our knowledge, since *E. coli* RNase HII is the only bacterial Type 2 RNase H enzyme tested on mismatched and damaged rNMPs, it remains unclear if other bacterial RNase HIIs can process the wide range of rNMP variants expected in genomic DNA.

In this study, we compare the activity of *E. coli* and *Bacillus subtilis* RNase HII on all four properly base‐paired rNMPs, mismatched rNMPs, and damaged rNMPs. We find that *E. coli* RNase HII is efficient at resolving properly base‐paired rAMP, rGMP, rCMP, and rUMP, as well as mismatched rNMPs, but shows differences in activity on rNMP lesions. Strikingly, while *B. subtilis* RNase HII efficiently cleaves rAMP, rUMP, and rCMP, the enzyme demonstrates poor processing of rGMP in a Watson–Crick base pair or mismatched context. We find that *B. subtilis* RNase HII is refractory to incision at rOH and r8oG, suggesting a substrate preference that more closely resembles eukaryotic RNase H2 than *E. coli* RNase HII. Together, our work highlights that bacterial RNase HII enzymes have different intrinsic capabilities for repairing rNMPs, a result that has important implications for how cells maintain genome integrity.

## 2 Results

### 2.1 EcoRNase HII Efficiently Incises All Four Canonical rNMPs

**FIGURE 1:**
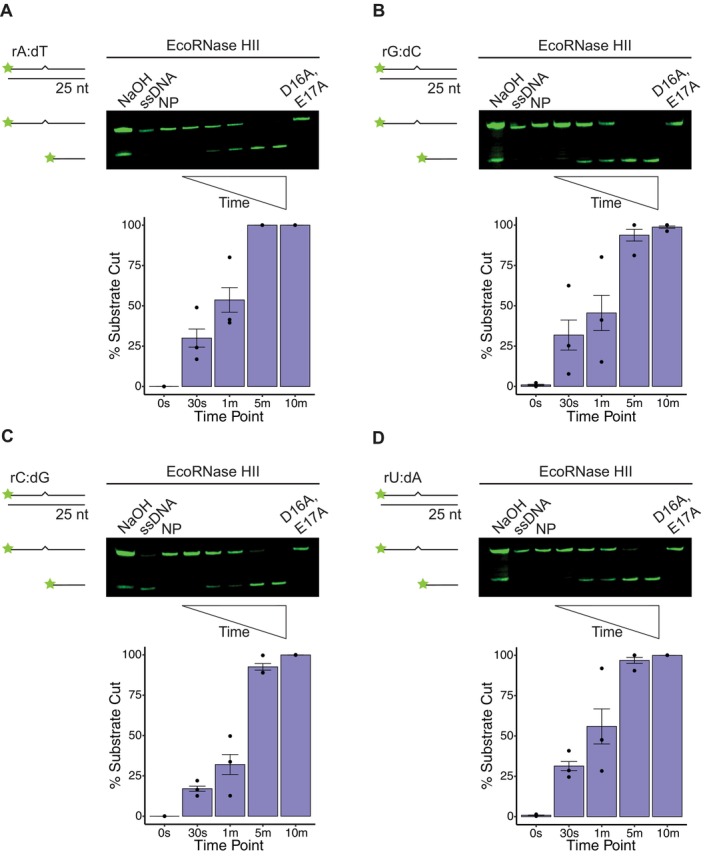
EcoRNase HII demonstrates similar nuclease activity on all four single rNMPs embedded in DNA. Representative denaturing urea‐PAGE for assays performed over 10 min at 37°C with 6.25 nM *E. coli* RNase HII and 100 nM of the following substrates: (A) rA:dT, (B) rG:dC, (C) rC:dG, and (D) rU:dA. Each substrate is composed of 25‐nucleotide long dsDNA with a single rNMP, represented by a zigzag. Quantification below each gel shows the mean percent of substrate incised over time (0 s, 30 s, 1 min, 5 min, and 10 min time points) with black bars to show the standard error for three replicates. Each gel contains ssDNA, no protein (NP), and catalytically impaired (D16A, E17A) controls. An alkaline ladder, represented by “NaOH”, was prepared by incubating substrate with 200 nM final NaOH. rA:dT was prepared with oJC3 and oJC2, rG:dC was prepared with oJC4 and oJC5, rC:dG was prepared with oJC6 and oJC7, and rU:dA was prepared with oJC8 and oJC9. Any difference observed for incision was not statistically significant.

*E. coli* RNase HII (EcoRNase HII) is the most well‐studied bacterial RNase HII enzyme (Friedberg et al. 2006). EcoRNase HII has been tested for activity on a variety of rNMP‐containing substrates in vitro (Ohtani et al. 2000; Rydberg and Game 2002; Malfatti et al. 2019; Friedberg et al. 2006). To our knowledge, EcoRNase HII activity has not yet been compared across all four canonical rNMPs in the same study (Itaya 1990; Ohtani et al. 2000; Haruki et al. 2002). Therefore, we overexpressed and purified EcoRNase HII from *E. coli* with a 6xHis‐SUMO tag. Following cleavage of the tag and further purification, we isolated EcoRNase HII that lacks any non‐native amino acids or tags (Experimental Procedures) (Figure S1). Our assays use a 25‐oligonucleotide dsDNA substrate containing rA:dT, rG:dC, rC:dG, or rU:dA. Each substrate is incubated with EcoRNase HII at 37°C, followed by electrophoresis using denaturing urea‐PAGE to resolve the fluorophore‐labeled rNMP‐containing strand. Denaturing conditions are used to allow for visualization of the strand break following cleavage by RNase HII (Randall et al. 2017; Schroeder et al. 2017; Ohtani et al. 2000). For assessment of each rNMP, we include a no protein control, a single‐stranded DNA (ssDNA) containing the rNMP, and a catalytically impaired EcoRNase HII protein variant (D16A, E17A), which has alanine mutations to the first two residues of the conserved DEDD active site (Rychlik et al. 2010). The substrate is also treated with NaOH to induce partial alkaline hydrolysis, generating a marker for both cleaved and uncleaved DNA. In the case of EcoRNase HII, rAMP, rGMP, rCMP, and rUMP show similar percentages of incision, with the reaction about 50% complete by 1 min and near 100% complete by 5 min (Figure 1). EcoRNase HII does appear slightly less efficient on rCMP with about 32% incised by 1 min (Figure 1C), although this difference was not statistically significant when compared to rAMP. We also tested the activity of RNase HII from at least two separate protein preparations and obtained the same results with the *E. coli* data shown in Figure S2. We conclude that EcoRNase HII incises all four rNMPs with comparable efficiencies.

Of note, we show that EcoRNase HII efficiently incises rUMP. Uracil DNA glycosylases (UDGs), which are involved in base excision repair (BER), recognize dUMP in DNA (Hayakawa et al. 1978; Lindahl 1974). UDGs can remove uracil from both ssDNA and dsDNA but not ssRNA due to steric clash of a conserved phenylalanine residue with the ribose sugar 2′‐OH (Pearl 2000; Savva et al. 1995). Our work shows that RNase HII efficiently incises rUMP, suggesting that this could be a main avenue for rUMP repair in vivo (see Discussion).

### 2.2 BsuRNase HII Inefficiently Incises rG:dC

**FIGURE 2:**
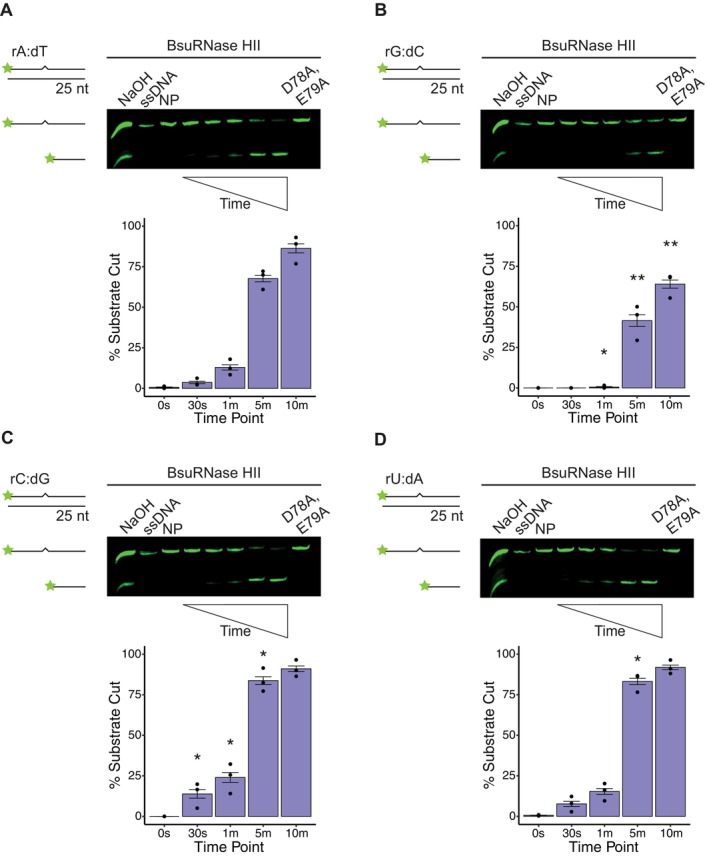
BsuRNase HII demonstrates weaker nuclease activity on rG:dC relative to other rNMPs. Representative denaturing urea‐PAGE for assays performed over 10 min at 37°C with 50 nM *B. subtilis* RNase HII and 100 nM of the following substrates: (A) rA:dT, (B) rG:dC, (C) rC:dG, and (D) rU:dA. Each substrate is composed of 25‐nucleotide long dsDNA with a single rNMP, represented by a zigzag. Quantification below each gel shows the mean percent of substrate incised over time (0 s, 30 s, 1 min, 5 min, and 10 min time points) with black bars to show the standard error for three replicates. Asterisks indicate significance, with * representing *p* < 0.05, ** representing *p* < 0.01, and *** representing *p* < 0.001 as compared to the same time point for rA:dT. Each gel contains ssDNA, no protein (NP), and catalytically impaired (D78A, E79A) controls. An alkaline ladder, represented by “NaOH”, was prepared by incubating substrate with 200 nM final NaOH. rA:dT was prepared with oJC3 and oJC2, rG:dC was prepared with oJC4 and oJC5, rC:dG was prepared with oJC6 and oJC7, and rU:dA was prepared with oJC8 and oJC9.

Next, we followed the same assay procedure as described for EcoRNase HII with *B. subtilis* RNase HII (BsuRNase HII). Like the *E. coli* protein, BsuRNase HII was purified after overexpression in *E. coli* using the 6xHis‐SUMO tag (Experimental Procedures) (Figure S1). As controls, we use a no protein, ssDNA containing the rNMP, and a catalytically impaired BsuRNase HII variant (D78A, E79A), where the first two residues of the conserved DEDD motif are mutated (Rychlik et al. 2010). An alkaline ladder was generated by incubating substrate with NaOH to induce partial hydrolysis of the substrate as described above. Like EcoRNase HII, BsuRNase HII has not been tested on all four canonical rNMPs in the same study (Itaya et al. 1999; Randall et al. 2017). Both EcoRNase HII and BsuRNase HII were tested in the same manner, although we used 50 nM BsuRNase HII as compared with 6.25 nM EcoRNase HII to achieve a similar percent of enzyme activity. Although there are moderate differences, we generally found that BsuRNase HII incised rAMP, rCMP, and rUMP with similar efficiencies, as each reaction reached roughly 14%–24% incision by 1 min (Figure 2A,C,D). For each substrate, we compared the percent incision at each time point to that for rAMP. We found a statistically significant increase in rCMP and rUMP incision for several time points relative to rAMP incision. Importantly, BsuRNase HII was very slow to incise rGMP, showing only ~0.5% incision by 1 min and less than 50% incision by 5 min (Figure 2B). Slow incision at rGMP was consistent among all the replicates, conditions, and independent protein preparations tested over the course of this study. We also found a statistically significant decrease in rGMP incision at three of the four time points collected, again relative to rAMP incision. As a control, we show that both EcoRNase HII and BsuRNase HII are unable to incise a rA:dT substrate in the absence of divalent metal (Figure S3). We conclude that BsuRNase HII is inefficient at cleaving rGMP in DNA, and like EcoRNase HII, is capable of efficient removal of rUMP from DNA, further supporting the model that rUMP is primarily targeted for repair by RER.

### 2.3 BsuRNase HII Inefficiently Incises Mismatched rGMP

**FIGURE 3:**
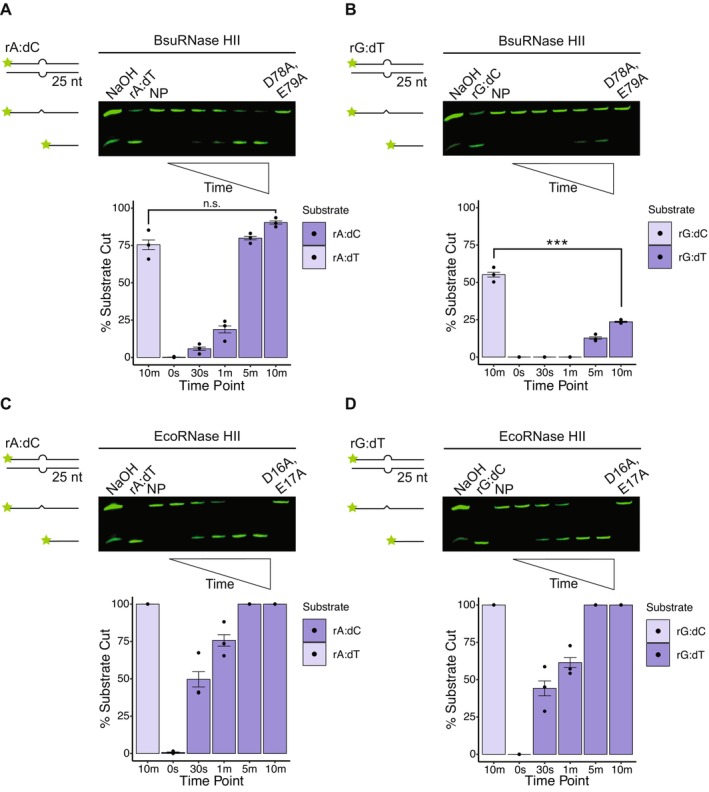
EcoRNase HII is more active on mismatched rNMPs than BsuRNase HII. Representative denaturing urea‐PAGE for assays performed over 10 min at 37°C with (A) 50 nM BsuRNase HII and 100 nM rA:dC, (B) 50 nM BsuRNase HII and 100 nM rG:dT, (C) 6.25 nM EcoRNase HII and 100 nM rA:dC, and (D) 6.25 nM EcoRNase HII and 100 nM rG:dT. Each substrate is composed of 25‐nucleotide long dsDNA with a single rNMP forming a mismatched base pair, which is represented by a bulge. Quantification below each gel shows the mean percent of substrate incised over time (0 s, 30 s, 1 min, 5 min, and 10 min time points) with black bars to show the standard error, for three replicates. Asterisks indicate significance, with * representing *p* < 0.05, ** representing *p* < 0.01, and *** representing *p* < 0.001 as compared to the same time point for rA:dT or rG:dC. Each gel contains positive (canonical base pair), no protein (NP), and catalytic impaired controls. An alkaline ladder, represented by “NaOH”, was prepared by incubating substrate with 200 nM final NaOH. rA:dC was prepared with oJC3 and oJC5, and rG:dT was prepared with oJC4 and oJC2.

Because rNMPs are the most frequent replication error made in vivo, it stands to reason that rNMPs could be mismatched, resulting in an error that could be recognized by RNase HII or MutS during MMR (Shen et al. 2011). Because dA:dC and dG:dT are the two most common mismatches formed in vivo (Kunkel 2004; Kunkel and Erie 2005; Lahue et al. 1989; Modrich and Lahue 1996), we chose to test BsuRNase HII on rA:dC and rG:dT under the hypothesis that these two rNMP mismatches are the most likely to occur during replication. We find that BsuRNase HII incises rA:dC as efficiently as the properly base‐paired ribonucleotides, suggesting that rA:dC could serve as a substrate in vivo (Figure 3). Consistent with our previous observation for rGMP in a Watson–Crick base pair, BsuRNase HII exhibits poor activity on mismatched rGMP (rG:dT). We compare the 10‐min time point of the matched rG:dC to mismatched rG:dT and find a significant decrease in processing. Thus, even though matched rGMP is processed inefficiently, BsuRNase HII is significantly worse at processing mismatched rGMP (Figure 3B). We use both a no protein control and catalytically impaired variant as a negative control, in addition to generating an alkaline ladder to mark the positions of cut and uncut DNA. Moreover, we also incubate BsuRNase HII in lane 2 of each gel with a canonical base pair (rA:dT or rG:dC) as an additional control to show the protein is active (Figure 3). Contrary to our observation for BsuRNase HII, we show that EcoRNase HII efficiently incises both mismatched ribonucleotides to nearly the same extent as properly base‐paired control ribonucleotides. Any observed difference in incision between the two mismatched substrates is not statistically significant when compared to incision of the corresponding matched substrates (Figure 3C,D). We conclude that BsuRNase HII recognizes mismatched rA:dC but is poor at recognition of mismatched rG:dT (see Discussion).

### 2.4 BsuRNase HII Is Refractory to Damaged Ribonucleotides In Vitro

**FIGURE 4:**
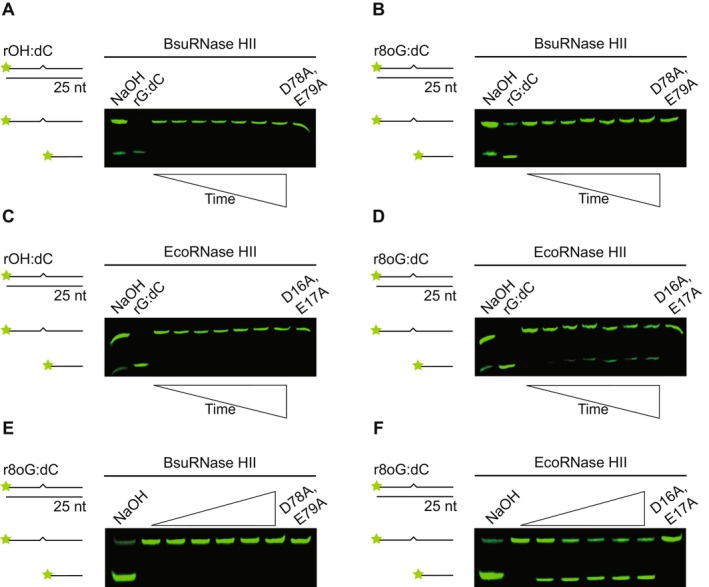
EcoRNase HII is more active on damaged rNMPs than BsuRNase HII. Representative denaturing urea‐PAGE for assays performed in triplicate over 160 min at 37°C. r8oxoG is abbreviated r8oG. The following conditions were used in (A) 50 nM BsuRNase HII and 100 nM rOH:dC, (B) 50 nM BsuRNase HII and 100 nM r8oG:dC, (C) 6.25 nM EcoRNase HII and 100 nM rOH:dC, and (D) 6.25 nM EcoRNase HII and 100 nM r8oG:dC. Gels in (E) and (F) show a comparison of incision at 160 min for increasing concentration of protein: 0, 6.25, 12.5, 25, 50, 100, and 100 nM catalytically impaired mutant. Each substrate is composed of 25‐nucleotide long dsDNA with a single rNMP, represented by a zigzag. In the case of rOH:dC, the sugar is intact, but the base is missing (abasic). The time points for all four gels are the same: 0 s, 5 min, 10 min, 20 min, 40 min, 80 min, and 160 min. Each gel contains positive (rG:dC) and catalytically impaired controls. An alkaline ladder, represented by “NaOH”, was prepared by incubating substrate with 200 nM final NaOH. rOH:dC was prepared with oJC10 and oJC5, and r8oG:dC was prepared with oJC11 and oJC5.

It has been shown previously that EcoRNase HII can incise damaged ribonucleotides, while eukaryotic RNase H2 orthologs cannot (Malfatti et al. 2019). We test BsuRNase HII on r8oG:dC and an abasic site with a ribose sugar, denoted as rOH:dC. We show that BsuRNase HII is refractory to incise both damaged ribonucleotides (Figure 4A,B). Additionally, upon increasing BsuRNase HII concentration, we still do not observe incision on r8oG:dC (Figure 4E). As a control, we show that BsuRNase HII cleaves an undamaged rNMP‐containing substrate (rG:dC) (Figure 4A,B). Alkaline ladder is generated with damaged substrate as described above to mark positions for cut and uncut DNA. We also test EcoRNase HII on the same damaged substrates. While we do not observe activity on the ribose‐containing abasic site (rOH:dC), we do observe some activity on the r8oG:dC (Figure 4C,D,F). Our results differ slightly from a prior report, which observed weak EcoRNase HII activity on rOH (Malfatti et al. 2017). Although our results are not identical to Malfatti et al. (2017), we do observe a similar trend in that r8oG serves as a reasonable substrate for EcoRNase HII, while rOH does not. We further test BsuRNase HII and EcoRNase HII on r8oG using a 2‐fold increase in Mg^2+^ to 2 mM, as well as 1 mM Mn^2+^. We do not observe BsuRNase HII incision of r8oG under either metal condition, while EcoRNase HII cleaves r8oG under both metal conditions (Figure S4). With our results, we conclude that BsuRNase HII is unable to incise damaged ribonucleotides, while EcoRNase HII can repair r8oG and not rOH. Further, with these results, we suggest that rOH sites would need to be repaired through BER (Wozniak and Simmons 2022). We conclude that rOH is either refractory to repair by RER or inefficiently repaired by RER in bacteria.

## 3 Discussion

It is well established that single ribonucleotide errors represent the most frequent non‐canonical nucleotide in need of repair (Williams et al. 2016; Zhou et al. 2021; Schroeder et al. 2015). Given that replicative and non‐replicative DNA polymerases make sugar errors at such a high frequency (Nick McElhinny, Watts, et al. 2010; Yao et al. 2013), it would be expected that mismatched and damaged rNMPs would be present in the genome and thus require repair. The increased likelihood of mismatched and damaged rNMPs in genomic DNA raises the question of how these complex errors are addressed. Our results show that bacterial RNase HII efficiently resolves mismatched rNMPs, while the activity observed for canonical and damaged rNMPs depends on the specific bacterial enzyme tested. These observations suggest that RNase HII substrate preference varies within the bacterial kingdom. This divergence in activity is especially interesting, as RNase HII is the only conserved RNase H family member between *E. coli* and *B. subtilis*, and it is the only RNase H conserved across all domains of life (Ohtani et al. 1999b).

The crystal structure of human RNase H2 shows that both an invariant tyrosine residue and a glycine‐arginine‐glycine (GRG) motif, together called the junction‐sensing module, form hydrogen bonds with the 2′‐OH of the ribose sugar (Rychlik et al. 2010; Chon et al. 2006; Lai et al. 2000). This tyrosine sets RNase HII apart from other family enzymes, like RNase HI or RNase HIII, which are unable to precisely recognize a single RNA–DNA junction (Nowotny et al. 2005; Nowotny and Yang 2006; Hyjek et al. 2019). Given the structural data, it seems that RNase HII would only require a 2′‐OH and an RNA–DNA junction to recognize all possible ribonucleotide variants (Rychlik et al. 2010; Chon et al. 2006; Lai et al. 2000). In most prior studies, a single rNMP has been chosen and studied as representative of all ribonucleotides cleaved by RNase HII. Given that each of the four rNMPs is incorporated into DNA at different rates (Yao et al. 2013; Balachander et al. 2020), we asked if bacterial RNase HII incises the four canonical rNMPs with similar efficiencies. We found that EcoRNase HII does indeed incise all four canonical rNMPs with similar efficiencies. Incision of rCMP is the least efficient, but overall, rAMP, rGMP, rCMP, and rUMP evoke similar processing by EcoRNase HII. Based on in vitro rNMP incorporation estimates, we would expect roughly 1533 rAMP, 331 rCMP, 124 rGMP, and 6 rUMP per round of replication for *E. coli* DNA polymerase III holoenzyme (Yao et al. 2013). Thus, even though rAMP is by far the most frequently incorporated rNMP by the *E. coli* replicase, EcoRNase HII incises all rNMPs equally well. In the case of BsuRNase HII, we consistently observe inefficient processing of rGMP, while rAMP, rCMP, and rUMP are processed with similar efficiencies. The reason for slow incision of rGMP by BsuRNase HII is unclear. *B. subtilis* is a low‐GC organism, although BsuRNase HII efficiently cleaves rCMP and rUMP, suggesting that the difference is not shaped by the abundance of particular rNMPs in the genome. Given that BsuRNase HII is so slow to process rGMP, we speculate that rGMP may persist in vivo, potentially leading to mutations and genome instability. It is also possible that other enzymes address rGMP in the *B. subtilis* genome. One candidate is the APE1 ortholog ExoA, which has been shown to cleave several nucleotide variants, including ribonucleotides embedded in DNA (Shida et al. 1999).

Another important finding from our work is that both *E. coli* and *B. subtilis* RNase HII process rUMP efficiently, which is impressive considering that we expect about 5–6 rUMP misincorporations per genome replication event (Yao et al. 2013). Although the number of rUMPs incorporated into the genome is expected to be low, another source of rUMP would be through deamination of rCMP to rUMP. Therefore, we suggest that rUMP is higher in abundance in the genome than anticipated as a result of both sugar errors by replicative DNA polymerases and spontaneous deamination events. Bacterial UDG is adept at removing dUMP from dsDNA and ssDNA, but it cannot use an ssRNA substrate (Pearl 2000; Savva et al. 1995). A recent paper found that UDG can remove a single rUMP from DNA in vitro; however, it does not appear to do so in vivo (Fan et al. 2025). Since UDG does not efficiently use an RNA substrate due to clash of a conserved phenylalanine residue with the 2′‐OH on the ribose sugar (Savva et al. 1995), rUMP would need to be repaired exclusively through RER. This would require that RNase HII removes rUMP formed through both DNA polymerase misincorporation events and deamination of rCMP to rUMP. Further, since uracil is mutagenic and results in GC‐to‐AT transitions (Duncan and Miller 1980; Duncan et al. 1978), RNase HII would help ensure fidelity during the replication process by removing rUMP.

**FIGURE 5:**
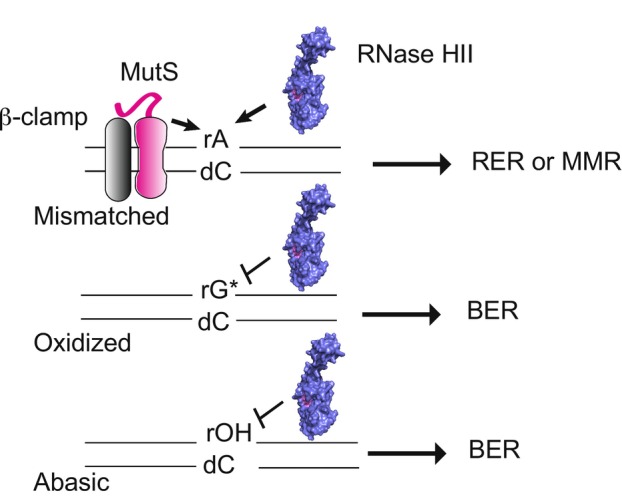
BsuRNase HII and mismatch repair correct rNMP mismatches while BER repairs damaged rNMPs. Shown is a model for the repair of mismatched and damaged rNMPs in *B. subtilis* . MutS, with the aid of β‐clamp (DnaN) and RNase HII, can bind and correct rNMP errors. Since RNase HII is inefficient at processing rG:dT mismatches, we propose that MutS corrects rG:dT, while both MutS and RNase HII correct rA:dC and other mispairs. Since BsuRNase HII is refractory to r8oG (rG*) and rOH, we propose that both are repaired through the BER pathway.

In the case of mismatched rNMPs, we test rA:dC and rG:dT because these ribonucleotide mismatches should be the most common mismatched rNMPs if rNMP errors follow the same trend as dNMP errors during replication (Kunkel 2004; Kunkel and Bebenek 2000; Kunkel and Erie 2005). EcoRNase HII is able to recognize both mismatches equally well, while BsuRNase HII efficiently incises rA:dC but not rG:dT. The fact that BsuRNase HII struggles to process rG:dT in a mismatch provides further validation that the *B. subtilis* enzyme is inefficient at processing rGMP. Prior work showed that EcoRNase HII was more active on mismatched rather than matched rNMPs (Malfatti et al. 2019). We also find that EcoRNase HII processes mismatched rNMPs more rapidly than those that are properly base‐paired. We speculate that when rAMP is involved in a mismatch, the rA:dC mispair may provide RNase HII with better recognition of the 2′‐OH. Given that bacterial RNase HII recognizes mismatched ribonucleotides quite well, we find it interesting that eukaryotic RNase H2 does not (Malfatti et al. 2019). We suggest that MMR addresses mismatched rNMPs in eukaryotes, whereas the bacterial MMR machinery is either poor at recognizing mismatched rNMPs or RER and MMR cooperate to correct mismatched rNMPs in the genome. Since bacterial MutS has not been tested on rNMP‐containing mismatches, we speculate that mismatched rNMPs are repaired by MMR and RER, providing two avenues for their correction (Figure 5).

In the case of damaged rNMPs, we provide clear evidence that BsuRNase HII does not repair rOH or r8oG. We also show that EcoRNase HII repairs r8oG but not rOH. Eukaryotic RNase H2 and archaeal RNase HII are refractory to incision of rOH and r8oG (Malfatti et al. 2017, 2019), and it has been suggested that recognition of damaged rNMPs has been lost through evolution from bacteria to eukaryotes (Malfatti et al. 2019). Like dNMPs, rNMPs are subject to oxidative damage and other modifications (Randerath et al. 1992; Loeb and Preston 1986). Our results show that the capacity of bacterial RNase HIIs to repair damaged rNMPs varies between *B. subtilis* and *E. coli*, two organisms separated by approximately two billion years of evolution (Pace et al. 1986). The ability of RNase H enzymes to recognize and repair damaged ribonucleotides may simply reflect the DNA repair repertoire of each organism tested. In the case of eukaryotes, APE1, not RNase H2, removes rOH and r8oG (Malfatti et al. 2017). For *E. coli* and *B. subtilis* , the role of RNase HII in repair of rOH and oxidative damage is less clear. EcoRNase HII can incise r8oG well, suggesting that EcoRNase HII and MutY may overlap to repair r8oG. Under the conditions tested here, EcoRNase HII and BsuRNase HII do not process rOH lesions, while BsuRNase HII does not repair r8oG. Based on our evidence, we conclude that rOH sites in both organisms and r8oG in *B. subtilis* would be repaired by BER (Figure 5).

## 4 Experimental Procedures

### 4.1 Plasmid Construction

A list of strains, plasmids, and primers used in this work is provided in Tables S1–S3, respectively. The *B. subtilis* PY79 *rnhB* gene (oJR88 and oJR89) and pE‐SUMO backbone (oJR46 and oJR47) were PCR amplified from purified JRC9 gDNA and HindIII‐digested pFCL1, respectively. These fragments were joined using Gibson Assembly to make pFCL25 (Gibson et al. 2009). Site‐directed mutagenesis of pFCL25 (oJR94 and oJR95) was performed to generate pFCL26. Similarly, the *E. coli* MC1061 *rnhB* gene (prJC36 and prJC40) and pE‐SUMO backbone (prJC44 and prJC45) were PCR amplified from purified JRC6 gDNA and HindIII‐digested pFCL25, respectively. The fragments were joined using Gibson Assembly to generate pJC10, which was subjected to site‐directed mutagenesis (prJC41 and prJC42) to make pJC11. The assembled plasmids were used to transform chemically competent *E. coli* MC1061, which was plated on LB agar with 25 μg/mL kanamycin (kan). Kanamycin‐resistant colonies were subjected to colony PCR ( *B. subtilis* : oJR88 and oJR89, *E. coli* : prJC36 and prFCL25), and plasmid sequences were verified via Sanger sequencing ( *B. subtilis* : prFCL25, *E. coli* : T7 and T7term) using Eurofins Scientific.

### 4.2 Protein Expression

Each expression plasmid was purified from the appropriate *E. coli* MC1061 strain and subsequently used to transform competent *E. coli* BL21(DE3) cells. Transformants were plated on LB agar (25 μg/mL kan) and incubated at 37°C overnight. A single colony was picked to inoculate 50 mL LB (25 μg/mL kan) and incubated at 37°C overnight while shaking at 200 rpm. 5–15 mL of this overnight culture were used to inoculate 1 L of LB (25 μg/mL kan) under sterile conditions. Scaled‐up cultures were grown at 37°C and 165–200 rpm until the OD_600_ reached between 0.5 and 0.7. Expression was induced with 0.5 mM isopropyl‐β‐D‐thiogalactopyranoside (IPTG) and incubated for 3 h at 37°C between 165 and 200 pm. Cells were harvested by centrifugation at 4000 rpm and 4°C for 30 min, after which each pellet was resuspended in 50 mL LB. Resuspended pellets were spun down again at 4000 rpm and 4°C for 30 min. Once the supernatant was decanted, the pellets were weighed, frozen, and then stored at −80°C until purification.

### 4.3 Protein Purification

Pellets were thawed on ice and resuspended in 30 mL Nickel Buffer A (20 mM Tris–HCl pH 8.0, 400 mM NaCl, 5% glycerol, and 1 mM DTT) using a serological pipet. After the addition of 1 EDTA‐free Pierce Protease Inhibitor tablet per pellet, the combined pellets were sonicated at 75% amplitude for an “on time” of 1 min and 40 s and spun down at 30,000 × *g* and 4°C for 40 min. Clarified lysate was loaded onto an equilibrated Ni‐NTA affinity column with a 5mL column volume (CV), and the flow‐through (FT) was collected. The column was washed with 5 CV of 100% Nickel Buffer A and 10 CV of 5% Nickel Buffer B (20 mM Tris–HCl pH 8.0, 400 mM NaCl, 5% glycerol, 1 mM DTT, and 300 mM imidazole); both washes were collected. The 6xHis‐SUMO‐tagged protein was eluted in 1mL fractions using 16 mL of 100% Nickel Buffer B. The concentration of each fraction was measured on a NanoDrop Microvolume Spectrophotometer, and those with an A_280_ close to or greater than 1 were pooled. Pooled fractions were diluted using Dilution Buffer (20 mM Tris–HCl pH 8.0, 5% glycerol, 1 mM EDTA, and 1 mM DTT) to reduce the salt to 300 mM and supplemented with 1 mM DTT. The pooled protein was then incubated with purified SUMO protease (Ulp1) for 2 h at room temperature. Post‐incubation, the protein was carefully pipetted into 10,000 MWCO dialysis tubing and dialyzed overnight at 4°C against Dialysis Buffer (20 mM Tris–HCl pH 8.0, 300 mM NaCl, and 5% glycerol) to remove imidazole and DTT.

The following morning, the dialyzed protein was filter‐sterilized using a 50‐mL syringe and a 0.22‐μM sterile filter that had been primed with Dialysis Buffer. The filter‐sterilized protein was added to the Ni‐NTA affinity column, which had been equilibrated with Dialysis Buffer and stored at 4°C the day before, and the FT was collected. Subsequently, 3 CV of filtered Dialysis Buffer were added to the column and collected as the Dialysis Wash. Finally, 3 CV of 5% Nickel Buffer B and 100% Nickel Buffer B were added sequentially to remove residual 6xHis‐SUMO tag and SUMO protease from the resin. SDS‐PAGE using a 10% polyacrylamide gel was used to verify the presence of pure protein in the filter‐sterilized protein FT and Dialysis Wash. These fractions were combined and concentrated using a 10,000 MWCO Amicon Ultra Centrifugal Filter. Glycerol was added to concentrated protein to 25% and the protein was flash frozen using liquid nitrogen before storage at −80°C.

Additional purification was performed using an ÄKTA FPLC. Both crude BsuRNase HII and EcoRNase HII were subjected to anion exchange chromatography using a HiTrap Q FF column (Cytiva 17505301) in Buffer A (20 mM Tris pH 8.0, 1 mM DTT, and 5% glycerol) and Buffer B (20 mM Tris pH 8.0, 1 mM DTT, 5% glycerol, and 500 mM NaCl). Sample, diluted to 50 mM NaCl in IEC Buffer A, was injected onto the column using a 50mL Superloop (Cytiva 18111382). The column was equilibrated with 10% IEC Buffer B, and protein was eluted via fractionation over a gradient of 100% IEC Buffer B. Peak fractions were identified using SDS‐PAGE, pooled, and concentrated using a 10,000 MWCO Amicon Ultra Centrifugal filter. EcoRNase HII and EcoRNase HII D16A, E17A were combined with glycerol to 25% and flash frozen using liquid nitrogen before storage at −80°C. A representative 10% SDS‐PAGE gel with 1 μg of each purified protein and stained with Coomassie blue dye is included (Figure S1).

BsuRNase HII and BsuRNase HII D78A, E79A were also purified via size exclusion chromatography using a HiPrep Sephacryl S‐200 HR preparative column (Cytiva 17116601) and Buffer A (20 mM Tris pH 8.0, final NaCl concentration of pooled protein from anion exchange, and 1 mM DTT). The column was equilibrated with Buffer A, and protein was eluted via fractionation. SDS‐PAGE was used to determine the peak fractions, which were then combined and concentrated as described above. Glycerol was added to 25%, and protein was flash frozen using liquid nitrogen before storage at −80°C. A representative 10% SDS‐PAGE gel with 1 μg of each purified protein and stained with Coomassie blue dye is included (Figure S1).

### 4.4 RNase HII Activity Assays

Assays performed herein were based upon a previously established method (Randall et al. 2019; Schroeder et al. 2017, 2023; Lowder and Simmons 2023).

RNA–DNA hybrid substrates were prepared by combining 1 μM 5′‐labeled rNMP‐containing oligonucleotide with 2 μM unlabeled complement in 1× TS Buffer (20 mM Tris pH 8.0, 100 mM NaCl), boiling at 98°C for 1 min, and cooling to anneal away from light. Oligonucleotides were used to assemble the following substrates: single canonical rNMP‐containing hybrids (oJC3:oJC2, oJC4:oJC5, oJC6:oJC7, and oJC8:oJC9), single mismatched rNMP‐containing hybrids (oJC3:oJC5 and oJC4:oJC2), and single damaged rNMP‐containing hybrids (oJC10:oJC5 and oJC11:oJC5). All oligonucleotides were 25 nucleotides long, and their sequences can be found in Table S4. Purified protein was diluted to a working concentration of 200 or 100 nM in 1× MgTS Buffer (20 mM Tris pH 8.0, 100 mM NaCl, 1 mM MgCl_2_). Reactions were prepared in 1× MgTS Buffer using 50 nM BsuRNase HII or 6.25 nM EcoRNase HII and 100 nM annealed substrate. Reactions were incubated on a heat block at 37°C, during which 8 μL time points were taken (0 s, 30 s, 1 min, 5 min, 10 min, unless otherwise noted), mixed 1:1 with 2× stop buffer (95% formamide, 20 mM EDTA, 0.01% bromophenol blue), boiled at 98°C for 5 min, and immediately stored on ice. The 0 s time point was prepared before starting the reaction by mixing diluted protein and reaction buffer in the appropriate ratio and immediately mixing with 2× stop buffer. Appropriate controls were performed depending on the nature of the assay, including no protein, catalytically impaired protein, ssDNA containing an rNMP, and/or a canonical hybrid substrate (in the case of the damaged rNMP assays, rG:dC). The ladder was prepared by incubating substrate with an equivalent amount of 400 nM NaOH at 37°C for 10 min, followed by boiling. Products were subjected to gel electrophoresis using 20% denaturing urea‐PAGE (8 M urea) and imaged using the 800 nm channel of a LiCor Odyssey imager. Three replicates were performed for each substrate.

### 4.5 Activity Assay Analysis

Analysis for activity assays has been previously described (Lowder and Simmons 2023; Randall et al. 2017; Schroeder et al. 2017). FIJI was used for the quantification of gel band intensities. The time point lanes were selected, using the 0 s lane as the reference. The intensities corresponding to each lane were plotted, and the Wand Tool was used to determine the area under the curve for each lane. Measurements from three replicates were exported to Microsoft Excel and used to determine the relative percent of cut substrate (area under curve for time point divided by area under curve for 0s time point, times 100). The following packages were used to process the data and generate graphs in R: *tidyverse*, *ggplot2*, and *forcats* (Wickham et al. 2016; Wickham et al. 2019).

### 4.6 Statistical Analysis

Statistical analyses were performed in R using the base package (R Core Team 2022). Using the lm() function, the data were fit to an ordinary least squares (OLS) linear regression model, where percent incision was treated as the response and substrate as the sole predictor. A separate model was generated for substrates at each time point (i.e., 10 min, etc.). For the BsuRNase HII assays with canonical rNMPs, all comparisons were made with rA:dT as the reference. Similarly, for BsuRNase HII assays with rA:dC and rG:dT, comparisons were made at 10 min only with rA:dT and rG:dC as the references, respectively. Using an alpha (⍺) of 0.05, significance is shown in each figure with * representing *p* < 0.05, ** representing *p* < 0.01, and *** representing *p* < 0.001.

## Author Contributions

**Julianna R. Cresti:** conceptualization, formal analysis, funding acquisition, investigation, validation, visualization, writing original draft, writing – review and editing. **Lyle A. Simmons:** conceptualization, formal analysis, funding acquisition, investigation, project administration, supervision, validation, visualization, writing original draft, writing – review and editing.

## Funding

This work was funded by National Institutes of Health (NIH) grant R35GM131772 to L.A.S. J.R.C. was supported by the following NIH training grant: Michigan Predoctoral Training in Genetics (T32GM007544). J.R.C. was also supported by precandidate and candidate research grants from the Horace H. Rackham Graduate School, in addition to two fellowships (Peter Olaus Okkelberg and Edwin H. Edwards) from the Department of Molecular, Cellular, and Developmental Biology at the University of Michigan.

## Ethics Statement

The cloning procedures in this work were approved by the Institutional Biosafety Committee (IBCA000000934).

## Conflicts of Interest

The authors declare no conflicts of interest.

## Supporting information


**Table S1:** Strains used in this study.
**Table S2:** Plasmids used in this study.
**Table S3:** Oligonucleotide primers used in this study.
**Table S4:** Oligonucleotides used in this study.
**Figure S1:** Purified BsuRNase HII and EcoRNase HII. SDS‐PAGE of purified BsuRNase HII and EcoRNase HII proteins used in this study. Lane 1 contains Bio‐Rad Precision Plus Protein Dual Color Standards (Bio‐Rad 1610374) with the corresponding molecular weight (MW) of each band indicated. Lanes 2–5 each contain 1 μg of *B. subtilis* RNase HII (28.4 kDa), *B. subtilis* RNase HII D78A E79A (28.3 kDa), *E. coli* RNase HII (21.5 kDa), and *E. coli* RNase HII D16A E17A (21.4 kDa), respectively.
**Figure S2:** Activity assessment of second EcoRNase HII purification.
(A) EcoRNase HII was purified a second time as described in “Experimental Procedures.” Shown is 1 μg of protein and the molecular weight (MW) standard (Bio‐Rad 1610374). (B) Representative denaturing urea‐PAGE for assays performed over 10 min at 37°C with 6.25 nM *E. coli* RNase HII. The substrate is composed of 25‐nucleotide long dsDNA with a single rNMP, represented by a zigzag. Quantification below each gel shows the mean percent of substrate incised over time (0 s, 30 s, 1 min, 5 min, and 10 min time points) with black bars to show the standard error for three replicates. The first lane contains an alkaline hydrolysis ladder to provide a marker for cut and uncut DNA. The second lane is ssDNA, followed by a no protein (NP) control. The last lane uses catalytically impaired (D16A, E17A) protein as a control. rG:dC was prepared with oJC4 and oJC5.
**Figure S3:** BsuRNase HII and EcoRNase HII incision is metal‐dependent. Shown are representative denaturing urea‐PAGE assays performed over a 160‐min time course (0 s, 5 min, 10 min, 20 min, 40 min, 80 min, 160 min time points) at 37°C with (A) 50 nM *B. subtilis* RNase HII and (B) 6.25 nM *E. coli* RNase HII. Assays were performed in the same manner as all other assays, with the exception that no divalent metal was added to the reactions. Each substrate is composed of a 25‐oligonucleotide dsDNA with a single rNMP, represented by a zigzag. Each gel contains ssDNA and catalytically impaired (D78A, E79A or D16A, E17A) controls, incubated for 160 min at 37°C. An alkaline ladder, represented by “NaOH”, was prepared by incubating substrate with 200 nM final NaOH. rA:dT was prepared using oligos oJC3 and oJC2.
**Figure S4:** Changing metal identity and concentration does not affect BsuRNase HII nuclease activity on r8oG:dC. Shown are representative denaturing urea‐PAGE assays performed over a 160‐min time course (0 s, 5 min, 10 min, 20 min, 40 min, 80 min, 160 min time points) at 37°C with (A) 50 nM *B. subtilis* RNase HII and 100 nM r8oG:dC, (B) 50 nM *B. subtilis* RNase HII and 100 nM r8oG:dC, (C) 6.25 nM *E. coli* RNase HII and 100 nM r8oG:dC, and (D) 6.25 nM *E. coli* RNase HII and 100 nM r8oG:dC. Assays shown in (A) and (C) were performed with 2 mM MgCl_2_, whereas (B) and (D) were performed with 1 mM MnCl_2_. Each substrate is composed of a 25‐oligonucleotide dsDNA with a single rNMP, represented by a zigzag. Each gel contains a canonical base pair (rG:dC) and catalytically impaired RNase HII as controls. An alkaline ladder was prepared by incubating each substrate with a final concentration of 200 nM NaOH. The rOH:dC substrate was prepared with oJC10 and oJC5, and r8oG:dC was prepared with oJC11 and oJC5.

## Data Availability

All data relevant to this manuscript are reported in the main or supporting information. Any other information is available upon request.
