# Supplementary material for: Bacillus subtilis RNase HII Is Inefficient at Processing Guanosine Monophosphate and Damaged Ribonucleotides
Source: Mol Microbiol. 2026 Jan 6;125(3):203–15. doi: 10.1111/mmi.70047 (PMC12956042; doi:10.1111/mmi.70047)
Supplement: Supplementary file 1 — Table S1: Strains used in this study. Table S2: Plasmids used in this study. Table S3: Oligonucleotide primers used in this study. Table S4: Oligonucleotides used in this study. Figure S1: Purified BsuRNase HII and EcoRNase HII. SDS‐PAGE of purified BsuRNase HII and EcoRNase HII proteins used in this study. Lane 1 contains Bio‐Rad Precision Plus Protein Dual Color Standards (Bio‐Rad 1610374) with the corresponding molecular weight (MW) of each band indicated. Lanes 2–5 each contain 1 μg of B. subtilis RNase HII (28.4 kDa), B. subtilis RNase HII D78A E79A (28.3 kDa), E. coli RNase HII (21.5 kDa), and E. coli RNase HII D16A E17A (21.4 kDa), respectively. Figure S2: Activity assessment of second EcoRNase HII purification. (A) EcoRNase HII was purified a second time as described in “Experimental Procedures.” Shown is 1 μg of protein and the molecular weight (MW) standard (Bio‐Rad 1610374). (B) Representative denaturing urea‐PAGE for assays performed over 10 min at 37°C with 6.25 nM E. coli RNase HII. The substrate is composed of 25‐nucleotide long dsDNA with a single rNMP, represented by a zigzag. Quantification below each gel shows the mean percent of substrate incised over time (0 s, 30 s, 1 min, 5 min, and 10 min time points) with black bars to show the standard error for three replicates. The first lane contains an alkaline hydrolysis ladder to provide a marker for cut and uncut DNA. The second lane is ssDNA, followed by a no protein (NP) control. The last lane uses catalytically impaired (D16A, E17A) protein as a control. rG:dC was prepared with oJC4 and oJC5. Figure S3: BsuRNase HII and EcoRNase HII incision is metal‐dependent. Shown are representative denaturing urea‐PAGE assays performed over a 160‐min time course (0 s, 5 min, 10 min, 20 min, 40 min, 80 min, 160 min time points) at 37°C with (A) 50 nM B. subtilis RNase HII and (B) 6.25 nM E. coli RNase HII. Assays were performed in the same manner as all other assays, with the exception that no divalent metal wa [file MMI-125-203-s001.docx]

Supplement Information

***Bacillus subtilis* RNase HII is inefficient at processing guanosine monophosphate and damaged ribonucleotides**

Julianna R. Cresti and Lyle A. Simmons^*^

Department of Molecular, Cellular, and Developmental Biology, University of Michigan, Ann Arbor, MI 48109

*LAS: Department of Molecular, Cellular, and Developmental Biology, University of Michigan, Ann Arbor, Michigan 48109-1055, United States. Phone: (734) 763-7142, Fax: (734) 647-0884 E-mail: [lasimm@umich.edu](mailto:lasimm@umich.edu)

**Short title:** RNase HII resolves canonical and mismatched ribonucleotides

**Keywords:** RNase HII, *Bacillus subtilis*, DNA replication, DNA damage, ribonucleotides, mismatches

**Supporting Table S1**. Strains used in this study.

| Strain ID | Species | Strain | Genotype/Plasmid | Reference |
| --- | --- | --- | --- | --- |
| FCL1 | *E. coli* | MC1061 | pFLC1 | Lab stock |
| FCL70 | *E. coli* | MC1061 | pFCL25 | Lab stock |
| FCL71 | *E. coli* | BL21(DE3) | pFCL25 | Lab stock |
| FCL72 | *E. coli* | MC1061 | pFCL26 | Lab stock |
| FCL73 | *E. coli* | BL21(DE3) | pFCL26 | Lab stock |
| JRC5 | *E. coli* | BL21(DE3) |  | Lab stock |
| JRC6 | *E. coli* | MC1061 |  | Lab stock |
| JRC9 | *B. subtilis* | PY79 | Prototroph Spβ° | (Youngman *et al.*, 1984) |
| JRC20 | *E. coli* | MC1061 | pJC10 | This work |
| JRC21 | *E. coli* | BL21(DE3) | pJC10 | This work |
| JRC22 | *E. coli* | MC1061 | pJC11 | This work |
| JRC23 | *E. coli* | BL21(DE3) | pJC11 | This work |

**Supporting Table S2.** Plasmids used in this study.

| Name | Genotype | Reference |
| --- | --- | --- |
| pFCL1 | His_6_-SUMO-*B. subtilis* *fenA* | This work |
| pFCL25 | His_6_-SUMO-*B. subtilis* *rnhB* | This work |
| pFCL26 | His_6_-SUMO-*B. subtilis* *rnhB* D78A E79A | This work |
| pJC10 | His_6_-SUMO-*E. coli* *rnhB* | This work |
| pJC11 | His_6_-SUMO-*E. coli* *rnhB* D16A E17A | This work |

**Supporting Table S3**. Oligonucleotides used as primers in this study.

| **Oligo** | **Sequence** |
| --- | --- |
| **oJR46** | tcgagcaccaccaccaccaccactgag |
| **oJR47** | acctccaatctgttcgcggtgagcctcaataatatcg |
| **oJR88** | ccgcgaacagattggaggtgtgaatacattaaccgtaaaggacattaaagacc |
| **oJR89** | tggtggtggtggtgctcgattatctgaaagattgaacaggagcg |
| **oJR92** | gtgaaatacttgggctgacagactc |
| **oJR94** | gttgccgcggtcggccg |
| **oJR95** | gaccgcggcaacacctgcaatc |
| **prFCL25** | cctttcgggctttgttagcagcc |
| **prJC36** | accgcgaacagattggaggtatgatcgaatttgtttatcc |
| **prJC40** | gtggtggtggtggtgctcgatcaggacgcaagtc |
| **prJC41** | tgtggctgcagtcggacgcgggccgttagttg |
| **prJC42** | ccgactgcagccacacccgcaaccagctgcg |
| **prJC44** | acctccaatctgttcgcggtg |
| **prJC45** | tcgagcaccaccaccaccac |
| **prJC51** | tccggcgtagaggatcgagatctcg |
| **prJC52** | cgggctttgttagcagccggatctc |

Primers were used for PCR amplification, mutagenesis, Gibson assembly, and colony PCR.

**Supporting Table S4**. Oligonucleotides used as substrates for RNase HII.

| **Oligo** | **Sequence** | **Label** |
| --- | --- | --- |
| **oJC1** | ggcttatacagcatcgagctcagga | 5ʹ IR800CWN |
| **oJC2** | tcctgagctcgatgctgtataagcc | n/a |
| **oJC3** | ggcttatacagcrAtcgagctcagga | 5ʹ IR800CWN |
| **oJC4** | ggcttatacagcrGtcgagctcagga | 5ʹ IR800CWN |
| **oJC5** | tcctgagctcgacgctgtataagcc | n/a |
| **oJC6** | ggcttatacagcrCtcgagctcagga | 5ʹ IR800CWN |
| **oJC7** | tcctgagctcgaggctgtataagcc | n/a |
| **oJC8** | ggcttatacagcrUtcgagctcagga | 5ʹ IR800CWN |
| **oJC9** | tcctgagctcgaagctgtataagcc | n/a |
| **oJC10** | ggcttatacagcrOHtcgagctcagga | 5ʹ IR800CWN |
| **oJC11** | ggcttatacagcr8-oGtcgagctcagga | 5ʹ IR800CWN |

Oligos were used to assemble double-stranded substrates for RNase HII endonuclease assays. All oligos are 25 nucleotides in length. The single rNMP modification has been

underlined for clarity.

**Figure S1**. **Changing metal and Mg^2+^ concentration does not affect BsuRNase HII nuclease activity on r8oG:dC.** Shown are representative urea-PAGE assays performed over a 160-minute time course at 37^o^C with (A) 50 nM *B. subtilis* RNase HII and 100 nM r8oG:dC, (B) 50 nM *B. subtilis* RNase HII and 100 nM r8oG:dC, (C) 6.25 nM *E. coli* RNase HII and 100 nM r8oG:dC, and (D) 6.25 nM *E. coli* RNase HII and 100 nM r8oG:dC. Assays shown in (A) and (C) were performed with 2 mM MgCl_2_, whereas (B) and (D) were performed with 1 mM MnCl_2_. Each substrate is composed of a 25-oligonucleotide dsDNA with a single rNMP, represented by a zigzag. Each gel contains a canonical base pair (rG:dC) and catalytic impaired RNase HII controls. An alkaline ladder was prepared by incubating each substrate with 200 nM NaOH. rOH:dC was prepared with oJC10 and oJC5, and r8oG:dC was prepared with oJC11 and oJC5.

**Figure S2.** **Purified BsuRNase HII and EcoRNase HII.** Shown is an SDS-PAGE. Lane 1 contains a protein dual color standards with the corresponding molecular weight (MW) indicated. Lanes 2-5 each contain 1 µg of *B. subtilis* RNase HII (28.4 kDa), *B. subtilis* RNase HII D78A E79A (28.3 kDa), *E. coli* RNase HII (21.5 kDa), and *E. coli* RNase HII D16A E17A (21.4 kDa), respectively.

**REFERENCES**

Youngman, P., Perkins, J.B., and Losick, R. (1984) Construction of a cloning site near one end of Tn917 into which foreign DNA may be inserted without affecting transposition in Bacillus subtilis or expression of the transposon-borne erm gene. *Plasmid* **12**: 1–9.
